# Interactions between Two Biological Control Agents on *Lygodium microphyllum*

**DOI:** 10.3390/insects9040180

**Published:** 2018-12-02

**Authors:** Ian Jones, Ellen C. Lake

**Affiliations:** 1 Department of Forestry, University of Toronto, 33 Willcocks Street, Toronto, ON M5S 3B3, Canada; 2 USDA-ARS Invasive Pant Research Laboratory, 3225 College Avenue, Fort Lauderdale, FL 33314, USA; ellen.lake@ars.usda.gov

**Keywords:** arthropod-plant interactions, oviposition behavior, Eriophyidae, insect-mite interactions, Lepidoptera

## Abstract

*Lygodium microphyllum* (Lygodiaceae) is an invasive climbing fern in peninsular Florida. Two classical biological control agents are currently being released against *L. microphyllum*: a leaf galling mite, *Floracarus perrepae* (Acariformes: Eriophyidae), and a moth, *Neomusotima conspurcatalis* (Lepidoptera: Crambidae). Little is known about how the two species interact in the field; thus we conducted oviposition choice tests to determine the effects of *F. perrepae* presence on oviposition behavior in *N. conspurcatalis*. Further, we conducted feeding trials with *N. conspurcatalis* larvae to establish the effects of gall presence on larval survival and rate of development, and determine whether *N. conspurcatalis* larvae would directly consume *F. perrepae* galls. *Neomusotima conspurcatalis* laid significantly more eggs on mite galled (52.66 ± 6.211) versus ungalled (34.40 ± 5.587) *L. microphyllum* foliage. Feeding trials revealed higher mortality in *N. conspurcatalis* larvae raised on galled (60%) versus ungalled (36%) *L. microphyllum* material. In gall feeding trials, *N. conspurcatalis* larvae consumed or damaged 13.52% of galls, and the rate of direct gall feeding increased over time as leaf resources were depleted. Our results suggest that, where *N. conspurcatalis* and *F. perrepae* co-occur, competitive interactions could be more frequent than previously anticipated; however, we do not expect these antagonistic interactions to affect the establishment of either agent.

## 1. Introduction

*Lygodium microphyllum* (Cavanilles) R Brown (Lygodiaceae), Old World climbing fern, is native to tropical and subtropical areas of Asia and Australasia and is an invasive fern in peninsular Florida [1]. First cultivated in the U.S. as an ornamental plant, the species was reported as naturalized in Florida in 1965 [2,3]. In recent years, the range of *L. microphyllum* in Florida has expanded dramatically [2,4,5], aided by its prolific production of microscopic, self-compatible spores that can travel large distances on wind currents [6,7]. *Lygodium microphyllum* is now widespread in wet and mesic environments throughout south and central Florida, covering nearly 800,000 ha [4,5,8]. The fern degrades habitats first by smothering native vegetation and reducing plant diversity. Second, the plant acts as a fire ladder, carrying fire into tree canopies that would not normally burn [9]. Conventional weed control methods like mechanical removal or the use of herbicides are expensive, damage non-target plants, do not offer a sustainable solution for the control of *L. microphyllum*, and can be challenging to apply to remote infestations [10].

Adding a biological control component to existing management options was desired to improve the potential for long term, sustainable control of *L. microphyllum*, and foreign exploration to find candidate agents began in Australia and southeast Asia in 1996 [10,11]. Two approved agents have established, and are currently being mass-reared and released against *L. microphyllum* in Florida as part of the Comprehensive Everglades Restoration Plan (CERP): a mite, *Floracarus perrepae* Knihinicki & Boczek (Acariformes: Eriophyidae), and the brown lygodium moth, *Neomusotima conspurcatalis* Warren (Lepidoptera: Crambidae) [12,13,14,15]. 

*Floracarus perrepae* feeds on subleaflets of *L. microphyllum*, causing the leaflet margins to roll, forming a gall with two to three tight windings [16]. The resulting galls act as domatia for the mites and are the site of oviposition and immature development [17]. The galls are diagnostic of mite presence in the field [11]. A single gravid female is capable of inducing gall formation; however, a mature gall may contain 50 or more mites [16]. The galls provide constant humidity for the mites, and relative humidity within the galls is presumed to be 100% [17]. Female *F. perrepae* prefer to oviposit on young, sterile leaflets of *L. microphyllum* [16]. Development from egg to adult takes approximately 7 days at 26 °C [17].

*Neomusotima conspurcatalis* is a small (11 mm wing-span) defoliating moth, native to southeast Asia and northern regions of Australia [18,19]. The moths complete their life cycle in 28 days at 25 °C. Females lay eggs in shingle-like clusters. Larvae feed on *L. microphyllum* foliage for four or, more typically, five instars, before pupating attached to the underside of leaflets or on rachises. *Neomusotima conspurcatalis* is multivoltine, completing 12–13 generations per year [20]. 

Releases of both biological control agents have been conducted in south and central Florida since 2008 and range expansion is ongoing [21,22]. In many sites *N. conspurcatalis* and *F. perrepae* have been released sympatrically. Across multiple sites and countries in their native range, *N. conspurcatalis* and *F. perrepae* have been observed to co-occur. *Neomusotima conspurcatalis* tends to prefer fertile fronds and *F. perrepae* galls are primarily found on sterile fronds, but field searching biases for collection purposes has prevented recording of detailed data of their interactions (Zonneveld, R. personal observation). It is, therefore, of critical importance to understand how frequently these agents will interact in the field, and whether interactions are competitive or synergistic. Because *F. perrepae* dispersal is mediated almost entirely by wind [17,21], and larval *N. conspurcatalis* have relatively low mobility, the extent of interactions between the two species will be determined largely by *N. conspurcatalis* oviposition behavior. 

Insects use a variety of visual, olfactory, and textural cues when choosing host plants. In large part, these choices are presumed to be made with the goal of maximizing larval performance [23]. Even within an individual host plant, several factors such as food quality [24], the presence of predators [25], and the presence of fungal endophytes [26] can influence the specific locations where insects choose to lay eggs. Females of some species tend to choose younger foliage for its high nutrient content and relative lack of mechanical defenses [24]. Additionally, ovipositing females sometimes show a preference for microhabitats with structural qualities that confer some physical protection to their developing offspring [27,28]. In the case of *N. conspurcatalis*, little is known about that factors that influence oviposition site choices within dense stands of *L. microphyllum*. Knowledge of these factors, however, is important for predicting the frequency and ways with which *N. conspurcatalis* and *F. perrepae* will interact in the field. Furthermore, an increased understanding of *N. conspurcatalis* oviposition behavior can inform agent release strategy, and contribute to greater levels of establishment in the field.

We conducted a series of laboratory experiments to determine the effects of *F. perrepae* induced galling on oviposition behavior by *N. conspurcatalis*. Further, we conducted no-choice feeding trials with *N. conspurcatalis* larvae, to establish the effects of gall presence on larval survival and rate of development. Additionally, we subjected galled *L. microphyllum* to *N. conspurcatalis* feeding to determine if larvae would consume *F. perrepae* galls, and if so, if the incidence of gall consumption would change over time with depletion of the host plant material. Finally, we assessed the effects of leaflet age on *N. conspurcatalis* oviposition behavior.

## 2. Materials and Methods

### 2.1. Insect Colonies

Insects were sourced from laboratory reared colonies of *N. conspurcatalis* that are regularly replaced with *N. conspurcatalis* collected from Florida field sites. Colony insects were fed on field collected *L. microphyllum* from a nearby infestation. All experiments were conducted using *L. microphyllum* grown in a shade house, in order to maintain consistent plant quality, and to ensure that all galling came from the same population of *F. perrepae*. All trials took place at the USDA-ARS Invasive Plant Research Laboratory in Fort Lauderdale, FL, USA. Laboratory conditions were held constant at 24 ± 1 °C, 35 ± 5% relative humidity, and 14 h light: 10 h dark throughout the experiments.

### 2.2. Plant Production

In the introduced range, *L. microphyllum* plants appear to vary in their susceptibility to galling by *F. perrepae* [14]. For this reason, two lines of *L. microphyllum* plants are being reared in shade houses at the USDA-ARS Invasive Plant Research Laboratory. The two lines have been produced from the spores of high susceptibility and low susceptibility *L. microphyllum* plants respectively. Because we maintain an active colony of *F. perrepae* at the USDA-ARS Invasive Plant Research Laboratory, a large percentage of the high susceptibility *L. microphyllum* plants on site tend to be infested by the mites. For this reason, all of the ungalled plants used in the experiments came from the low susceptibility line, while all of the galled plants came from the high susceptibility line. All experimental plants were kept in 3-gallon pots, and were subjected to the same light, watering, and fertilizer treatments. All leaf material used for feeding trials were sourced from five high susceptibility and five low susceptibility *L. microphyllum* plants. *Neomusotima conspurcatalis* eggs, larvae, and pupae were manually removed from the plants as needed, and all food material was examined for eggs prior to inclusion in the experiments. 

### 2.3. Oviposition Choice Tests 

We tested whether the *F. perrepae* galls affected *N. conspurcatalis* oviposition choice by presenting gravid female *N. conspurcatalis* with galled and ungalled material. All oviposition trials were conducted in 2017. Individual moths were sexed as pupae, by examining the abdomen for the elongated genital pore on the females [20]. We set up 15 × 18 × 40 cm oviposition boxes (Pioneer Plastics, North Dixon, KY, USA) containing one female and three male *N. conspurcatalis* pupae, which were placed in the boxes prior to emergence. Each oviposition box contained two approximately equal sized ‘sprigs’ of *L. microphyllum*, one of which was heavily galled by the mite, *F. perrepae*, and the other one was free of galling. Sprigs consisted of a single pair of opposite pinnules connected to 8–10 cm of rachis material, placed in a water pick to prevent desiccation. Females were allowed to oviposit for 5 days after emergence, and sprigs were changed on the third day. *Neomusotima conspurcatalis* females oviposit half of their total eggs on the first night after mating; thus, the majority of a female’s lifetime oviposition occurred during this 5-day period [20]. We alternated the position of the galled and ungalled sprigs between replicates, and switched them within replicates when new sprigs were introduced, in order to control for any positional biases. After 5 days, we examined sprig material under a dissecting microscope and recorded the number of egg clusters, and the total number of eggs, on galled and ungalled sprigs. The proximity of egg clusters to galls was also assessed. Egg clusters were deemed to be under or adjacent to galls if they were separated from the gall by a distance smaller than the shortest width of the cluster. We set up a total of 36 oviposition boxes. Replicates in which no oviposition was observed (6) were removed from the analysis. 

To test the effects of leaflet age on *N. conspurcatalis* oviposition we set up oviposition boxes, as above, presenting the moths with a choice between young and old ungalled *L. microphyllum* sprigs. Young and old sprigs, within an individual oviposition box, were sourced from a single *L. microphyllum* rachis. The youngest pair of fully expanded pinnules (closest to the apical meristem) was used as the ’young’ sprig, and a pair of opposite pinnules between 8 and 10 nodes down the rachis was used as the ‘old’ sprig. We set up a total of 33 oviposition boxes. Three replicates, in which no oviposition was observed, were removed from the analysis.

### 2.4. Larval Feeding Trials

We tested whether the presence of *F. perrepae* galls affected larval development of *N. conspurcatalis*. Bouquets of 9–12 sprigs of galled and ungalled *L. microphyllum* material were placed separately into two oviposition cages each containing 110 *N. conspurcatalis* adults, at least 70 were newly emerged, the remainder were 2 days old. After 24 h bouquets, now bearing numerous egg clusters, were removed from the cages and placed in 10 × 15 × 25 cm clear plastic display boxes (Pioneer Plastics, North Dixon, KY, USA). Once larvae emerged and began feeding, the original oviposition sprigs were supplemented with additional food.

On 24 July 2018, approximately second instar larvae were transferred using a paintbrush from the communal feeding containers to quart-sized plastic soup containers with a sprig of leaf material in a large water pic supported by a vial. Leaf material consisted of at least one pinnule but more typically a pair of opposite pinnules. The majority of leaflets on galled sprigs had been galled by *F. perrepae*. Twenty-four hours after transfer to the individual sprigs the larvae were checked for mortality. Any larval death during this window was attributed to handling mortality and these individuals were excluded from the experiment. Sprigs were changed as needed to ensure a constant supply of high quality leaf material. For each larva, we recorded the time to pupation, pupal weight, and time to eclosure. Pupae were weighed once fully sclerotized to minimize handling mortality, typically within 48 to 72 h after forming. Pupae were also sexed as female *N. conspurcatalis* tend to be larger than males [22]. Where mortality occurred, developmental stage at death was recorded.

### 2.5. Consumption of Galls by N. conspurcatalis

We conducted an additional experiment to quantify whether *N. conspurcatalis* directly fed on galls or avoided galls during larval development. Twenty sprigs with the majority of leaflets galled were collected from the same five galled *L. microphyllum* plants used in the larval development experiment. The sprigs were checked for *N. conspurcatalis* eggs and larvae and the underside of each sprig was photographed to record the total number of galls and location of each gall for all sprigs. On 7 August 2018, the sprigs were placed in a water pic inside a quart container using the methods described above and one approximately third instar larva was added to each sprig. The sprigs were checked five times over the next week and the number of galls with new *N. conspurcatalis* feeding damage and the location of damage was recorded. Sprigs were not changed in order to simulate the depletion of food resources. The date each larva pupated was recorded.

### 2.6. Statistical Analysis

For the two oviposition experiments, the number of egg clusters, the total number of eggs laid, and the average number of eggs per cluster on galled vs. ungalled *L. microphyllum* sprigs were compared using two-tailed paired *t*-tests. 

For the larval feeding experiment, the effects of leaflet type (galled and ungalled) on survival to pupation and adult emergence was compared using Chi-square tests. Larval development time of individuals feeding on galled and ungalled *L. micropyllum* was compared using non-parametric Mann-Whitney U tests, as data were not normally distributed even after transformation. The effects of leaflet type consumed on pupal weight by sex was compared using a two-way ANOVA. All statistical tests were performed in SPSS version 24 (IBM, Armonk, NY, USA).

Data from the direct gall consumption experiment were zero inflated, and were not normally distributed even after transformation. To determine if the rate of direct feeding on galls, by *N. conspurcatalis* larvae, changed over time we conducted a non-parametric Friedman’s ANOVA. We then conducted Wilcoxon tests for pairwise comparisons between observation periods.

## 3. Results

### 3.1. Oviposition Choice Tests

**Figure 1:**
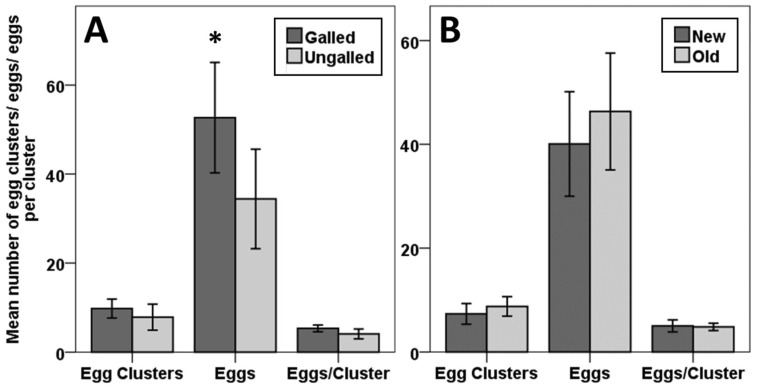
Mean numbers of egg clusters, individual eggs, and the mean number of eggs per cluster, laid by a single *N. conspurcatalis* female on (**A**) galled and ungalled *L. microphyllum* sprigs (*n* = 30), and (**B**) young and old *L. microphyllum* sprigs (*n* = 30). Error bars indicate standard error. Asterisks indicate significant differences.

*Neomusotima conspurcatalis* preferentially oviposited on galled material, but tended not to lay in close proximity to the galls themselves. The mean number of egg clusters laid by female *Neomusotima conspurcatalis* was not significantly different between galled and ungalled *Lygodium microphyllum* sprigs (t = −1.002, df = 29, SE = 1.928, *p* = 0.324). The mean number of eggs laid by *N. conspurcatalis* was, however, significantly higher on galled (52.66, SE = 6.211) vs. ungalled (34.40, SE = 5.587) sprigs (t = −2.214, df = 29, SE = 8.25, *p* = 0.035). The mean number off eggs per cluster was close to significantly higher on galled leaf material (5.34, SE = 0.381) than it was on ungalled leaf material (4.09, SE = 0.553) (t = −2.037, df = 29, SE 0.616, *p* = 0.051) (Figure 1A). Of 1580 eggs laid on galled leaf material, only 311 (19.68%) were laid next to or underneath galls. Of the 2612 eggs laid throughout the study, 2440 (93.42%) were laid on the underside of leaves.

We found no effects of leaf age on *N. conspuracatalis* oviposition. The mean number of egg clusters laid by female *N. conspurcatalis* was not significantly different between new (7.33, SE = 0.995) and old (8.76, SE = 0.936) *L. microphyllum* leaf material (t = −0.975, df = 29, SE = 1.47, *p* = 0.338). We also observed no difference in the mean number of eggs laid by *N. conspurcatalis* between new (40.06, SE = 5.04) and old (46.33, SE = 5.636) *L. microphyllum* sprigs (t = −0.790, df = 29, SE = 7.936, *p* = 0.436). The mean number off eggs per cluster was not significantly different between new (5.02, SE = 0.577) and old (4.83, SE = 0.355) leaf material (t = 0.295, df = 29, SE = 0.628, *p* = 0.771) (Figure 1B). Of 2592 eggs laid throughout the study, 2574 (99.30%) were laid on the underside of leaves.

### 3.2. Larval Feeding Trials

**Figure 2:**
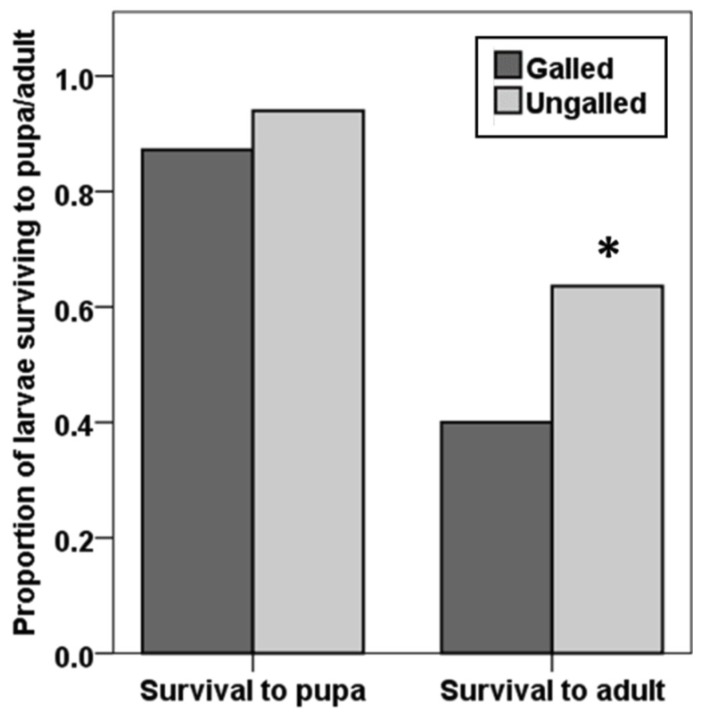
Proportion of *N. conspurcatalis* larvae, feeding on galled or ungalled *L. mocrophyllum* sprigs, surviving to pupation and adulthood (*n* = 110). Asterisks indicate significant differences.

*Neomusotima conspurcatalis* larvae reared on different *L. microphyllum* leaflet types (galled and ungalled) were equally likely to survive to pupation (Χ^2^ = 1.760, df = 1, *p* = 0.185); however, development to adult eclosure was significantly lower in individuals reared on galled *L. microphyllum* (40%) compared with those reared on ungalled *L. microphyllum* (63.6%) (Χ^2^ = 6.154, df = 1, *p* = 0.013) (Figure 2). We observed no differences in development time from egg to pupa (*n* = 100, Z = −0.214, df = 98, *p* = 0.831) or from egg to adult (*n* = 57, Z = −1.321, df = 55, *p* = 0.186) among *N. conspurcatalis* individuals reared on galled and ungalled *L. microphyllum*.

Leaflet type consumed (galled and ungalled) had no effect on pupal weight (*n* = 100, F = 0.101, df = 1, *p* = 0.751), and sex ratios were not different between treatments (*n* = 100, Χ^2^ = 0.174, df = 1, *p* = 0.677). Pupal weight was affected by sex (*n* = 100, F = 40.934, df = 1, *p* = 0.000), with female pupae ($\bar{x}$ = 6.9617 mg, SE = 0.173) weighing significantly more than male pupae ($\bar{x}$ = 5.5406 mg, SE = 0.14).

### 3.3. Consumption of Galls by N. conspurcatalis

**Figure 3:**
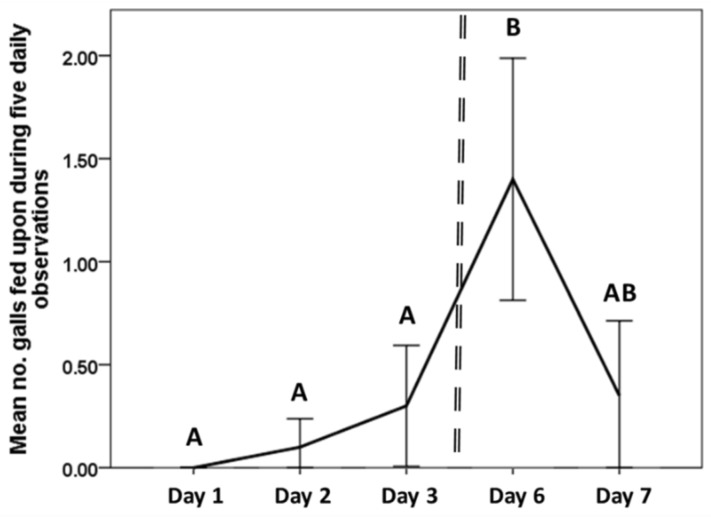
The number of new galls fed upon by individual 3rd/4th instar *N. conspurcatalis* larvae (*n* = 20) over 5 observation days. The line represents the mean number of galls fed upon over each sample period. The dashed vertical line represents a break in data collection between days 3 and 6. Error bars represent standard error. Different letters indicate significant differences.

We observed direct consumption of galls, albeit consumption was relatively rare, occurring mainly after high quality feeding sites had been exhausted. The 20 *L. microphyllum* sprigs used for the direct gall feeding experiments contained a total of 318 galls, of which 43 sustained direct feeding damage. In addition to this, in four of the replicates at least one gall was left dangling from the plant, or completely removed, as a result of adjacent feeding. Incidences of direct gall feeding by *N. conspurcatalis* increased significantly over the course of the experiment (*n* = 20, Χ^2^ = 30.478, df = 4, *p* = < 0.001). More galls were consumed on day 4 of the experiment than on days 1 (Z = −3.209, *p* = 0.001), 2 (Z = −3.213, *p* = 0.001) and 3 (Z = −2.898, *p* = 0.004) (Figure 3).

## 4. Discussion

*Neomusotima conspurcatalis* females showed a preference for ovipositing on galled *Lygodium microphyllum* material, but tended to avoid laying eggs close to the galls themselves. Larval development times were similar on galled and ungalled material, however, the proportion of individuals surviving to adult eclosure was lower on galled material. Direct feeding on galls was relatively rare, and the rate of gall feeding seemed to increase over time, as preferred feeding sites became scarce. Overall, we present mixed evidence for antagonistic interactions between *N. conspurcatalis* and *F. perrepae*.

Many weed biological control programs involve the release of multiple agents [29]. This is informed, in part, by the multiple stress hypothesis which suggests that plants require more than one stressor in order to be effectively suppressed [29]. In many cases, the assumption has been made that agents feeding in a complementary manner (i.e.: agents feeding on different plant organs or agents from different feeding guilds) would not compete because they do not interact directly [30]. This assumption, however, requires greater scrutiny. Indeed, the plethora of ways in which biological control agents interact, indirectly, through their effects on the host plant are reviewed by Milbrath and Nechols [31]. These indirect interactions by herbivores include eliciting changes in plant chemistry, nutritional quality, and morphology [32], as well as influencing the number and activity of natural enemies [33]. Of even greater concern, perhaps, are direct interactions between herbivores, competing for preferred feeding sites, which may affect the performance of one or both species [34]. In the case of the biological control program for *L. microphyllum*, we have sought to understand agent interactions through a series of laboratory oviposition and feeding experiments. 

During oviposition choice tests, *N. conspurcatalis* females showed a significant preference for galled *L. microphyllum* leaf material (Figure 1A). These results are surprising as studies have demonstrated that some herbivorous insects, particularly Lepidoptera, can be repelled by damage-induced plant volatiles [34], a behavior thought to aid herbivores in the avoidance of competition [35]. While the production of volatile organic compounds (VOCs) in response to herbivory is well studied in higher plants, relatively few studies have explored the phenomenon in ferns. One such study with the bracken fern, *Pteridium aquilinum*, determined that VOC production was not induced by damage from two defoliating herbivores [36]. Future work should explore the production of VOCs by *L. microphyllum* in response to damage caused by different feeding guilds, as well as their effects on the behavior of the biological control agents and their natural enemies. 

While the preference of *N. conspurcatalis* for galled leaf material was clear, the ecological explanation remains less obvious. Galled *L. microphyllum* leaflets are more structurally complex than ungalled leaflets. One possibility is that this structural complexity confers some degree of physical protection/shelter for developing eggs and emerging larvae [28]. Herbivorous Lepidoptera have previously been observed to make oviposition choices based on the presence of shelters. For example, shelters made by the birch tube maker, *Acrobasis betulella*, are regularly colonized by later generations of the moth (and by other moth species) through oviposition. This occurs equally readily in artificially constructed shelters, indicating that ovipositing females are responding to the presence of the shelter, and not chemical cues from the insects or the plant [27]. Positioning eggs under or adjacent to galls could potentially reduce the access of parasitoids or predators to part or all of the egg mass; *N. conspurcatalis* eggs are regularly parasitized by a trichogrammatid wasp [37]. Previous oviposition experiments have shown that *N. conspurcatalis* preferentially oviposits on fertile leaflets of *L. microphyllum* [22], which are more structurally complex than sterile leaflets due to finger-like projections bearing sori [4]. This lends some support to the idea of structural protection as an important factor in *N. conspurcatalis* oviposition. However, in the present study, while significantly more eggs were laid on galled leaf material, only 19.68% of eggs were found under, or adjacent to, the galls themselves. Our results, therefore, provide little support for this theory.

A possible alternative explanation for the observed preference of *N. conspurcatalis* for galled leaf material was that our results may have been skewed by a confounding factor, leaflet age. In order to limit complications from females laying eggs on ungalled parts of galled pinnules, we chose heavily galled pinnules for use in oviposition tests. Because it takes time for pinnules to develop that level of galling, the galled sprigs we used in the experiment tended to be at least 2 weeks old. Although we tried to control for pinnule age by selecting ungalled pinnules of a similar age, it seemed possible that leaflet age might be contributing to the preference shown by *N. conspurcatalis* for galled sprigs. Indeed, many fast growing plants like *L. microphyllum* concentrate defensive chemicals in young leaves [38,39]. In order to test this idea, an additional set of oviposition choice tests was conducted, comparing oviposition by *N. conspurcatalis* females on young versus old ungalled *L. microphyllum* sprigs. No preference was observed among *N. conspurcatalis* females for older vs. younger pinnules (Figure 1B). These results suggest two things. First, that *N. conspurcatalis* females are not deterred by any potential changes in defensive chemistry that occur as *L. microphyllum* leaflets age and, second, that the preference shown by *N. conspurcatalis* to lay eggs on *F. perrepae* galled leaflets was not driven by their presence on older leaf material.

One inevitable outcome of the observed oviposition and feeding behavior is that where the two biological control agents co-occur, particularly if both agents are present in high densities, larvae of *N. conspurcatalis* may feed on *F. perrepae* infested *L. microphyllum*. Larval feeding trials indicated that while larval development times were not affected by the presence of galls, adult eclosure of larvae feeding on galled material was significantly reduced. Eriophyid mites are known to alter the quality of infested plant tissues and, consequently, the behavior and fitness of other herbivores [40]. Croft and Hoying [41] determined that apple trees infested early in the season by the eriophyid mite, *Aculus schlechtendali*, were less likely to support populations of the red spider mite, *Panonychus ulmi*. Conversely, infestations of *Aculops* spp. on willow trees do not affect larval survival in the gall midge, *Dasineura marginemtorquens* [42]. While the effects of *F. perrepae* on *L. microphyllum* leaf chemistry are not known, it should be noted that the growth habit and rate of *L. microphyllum* in its invasive range, will likely reduce the rate of interactions between *N. conspurcatalis* and *F. perrepae*. *Lygodium microphyllum* typically grows in dense mats or tree skirts with intertwined rachises. Even in heavily galled sites, not all rachises contain galled material. Thus, larvae of *N. conspurcatalis* would likely not have to move far within foliage to find non-galled leaf material. Additionally, the *L. microphyllum* present in Florida differs in its susceptibility to *F. perrepae*, which is presumed to limit field establishment of the mite and would further reduce interactions between the two agents [14].

Previous oviposition experiments have demonstrated that *N. conspurcatalis* preferentially oviposits on fertile leaflets of *L. microphyllum* [22]. While this preference may be driven by a reduction in a potential defensive compound, 1-octen-3-ol in fertile leaflets, or the selection of a more protected oviposition sites, it has also been suggested that this behavior might be the result of niche-partitioning among *Lygodium* herbivores [22]. Indeed, *F. perrepae* has been observed to prefer sterile leaflets of *L. microphyllum* [11,17]. While it is possible that some niche partitioning may occur, our results suggest that direct competition for resources between *N. conspurcatalis* and *F. perrepae* might occur more frequently than we had expected. It is unknown whether these interactions would be extensive enough to limit the establishment or population growth of either agent given the abundance of *L. microphyllum* in invaded sites. Indeed, the two agents co-occur at relatively high densities at several Florida field sites. Future work should seek to compare the establishment of *N. conspurcatalis* in Florida field sites in the presence and absence of *F. perrepae*. 

The gall feeding experiments showed that direct feeding by *N. conspurcatalis* on *F. perrepae* galls was relatively rare, and suggested that gall feeding by *N. conspurcatalis* is likely incidental rather than active. Nevertheless, some galls were partially or completely removed from plants by larval feeding, and this larval feeding on galls increased over the course of the experiment (Figure 3), suggesting that galls represent non-optimal forage for *N. conspuratalis* and were consumed as higher quality feeding sites became more scarce. The rate of gall feeding by *N. conspurcatalis* actually decreased on the final observation day, however, by this stage of the experiment, seven of the 20 larvae had either pupated, or were in the process of pupating. Larvae often fed right up to the margin of a gall, sometimes leaving the gall hanging from the leaflet, likely causing desiccation. While galls do not appear to represent priority feeding sites, the activity of *N. conspurcatalis* could, nevertheless, affect *F. perrepae* populations negatively. Quantifying the effects of *N. conspurcatalis* on the populations of *F. perrepae* in potted *L. microphyllum* plants might represent a realistic next step. Additional laboratory bioassays could evaluate *N. conspurcatalis* larval behavior when given a choice between galled and non-galled *L. microphyllum*. 

## 5. Conclusions

Little is known about how interactions between *Neomusotima conspurcatalis* and *Floracarus perrepae* might affect populations of either agent in their introduced range. Our observation that *N. conspurcatalis* preferentially oviposits on galled *L. microphyllum* material suggests that direct interactions between these two agents could be relatively frequent where they co-occur; however, it remains unclear if these interactions will have negative consequences for either agent. Given the current distribution of the two agents, and the abundance of *L. microphyllum* in the introduced range, potentially negative interactions are not expected to limit the biological control program. 

Direct and indirect interactions among herbivores are common, but are often overlooked in the context of weed biological control, but see Reference [31]. Addressing these interactions can help to improve decision-making on the suitability of candidate biological control agents, aid agent establishment, and maximize weed suppression.
